# Cocaine- and amphetamine-regulated transcript (CART) peptide-positive neuron populations in the enteric nervous system of the porcine descending colon depend on age and gender

**DOI:** 10.1371/journal.pone.0321339

**Published:** 2025-04-04

**Authors:** Krystyna Makowska, Kainã R. C. Fagundes, Renata de Britto Mari, Sławomir Gonkowski

**Affiliations:** 1 Department of Clinical Diagnostics, Faculty of Veterinary Medicine, University of Warmia and Mazury in Olsztyn, Olsztyn, Poland; 2 Institute of Biosciences – Coastal Campus, São Paulo State University (Unesp), São Paulo, Brasil; 3 Department of Clinical Physiology, Faculty of Veterinary Medicine, University of Warmia and Mazury in Olsztyn, Olsztyn, Poland; University of Texas Medical Branch at Galveston, UNITED STATES OF AMERICA

## Abstract

The enteric nervous system (ENS) is a complex structure located in the wall of the gastrointestinal tract. One of the less-known active substances found in the ENS is cocaine- and amphetamine-regulated transcript peptide (CART). It is known that CART-positive enteric neurons take part in the reactions to pathological stimuli, but knowledge of physiological stimuli-dependent changes in their population is extremely limited. The aim of the present study was to investigate the age- and gender-dependent diversities in the distribution of CART-positive neurons in the porcine colonic ENS using the double immunofluorescence technique. The obtained results have shown that age affects the number of CART-positive neurons in the colonic ENS and the character and intensity of age-caused changes depend on the type of the enteric plexus, and the most visible changes have been noted in the myenteric plexus in which the percentage of CART-positive neurons amounted to 22.3 ± 0.2% in young females, 20.7 ± 0.4% in young males, 23.7 ± 0.2% in adult females and 25.8 ± 01% in adult males. Moreover, during the present study, sex-dependent diversities in the percentage of CART-positive neurons were found, especially in adult animals. The obtained results suggest that CART in the ENS takes part in neuroplasticity processes occurring during the development, maturation and/or aging of the gastrointestinal tract, as well as that the number of CART-positive neurons is controlled by sex hormones and depends on the gender. However, the elucidation of all aspects connected with the influence of age and gender on the population of CART-positive neurons in the ENS requires further comprehensive studies.

## Introduction

The enteric nervous system (ENS) which is situated in the wall of the gastrointestinal tract. takes part in the regulation of many gastric and intestinal functions [1,2]. Due to the millions of neurons constituting the ENS and its high degree of independence from the central nervous system (CNS), it is often called the “second” or “gut brain” [3–5].

The enteric neurons form intramural ganglia and are often interconnected with each other by a dense network of fibres and create ganglionated plexuses [6–10]. In the large intestine of the domestic pig, the ENS is built of three types of enteric plexuses: 1) myenteric plexus (MP) located between circular and longitudinal muscular fibres; 2) outer submucous plexus (OSP) – located in the submucosal layer, laid over the circular muscle fibres and 3) inner submucous plexus (ISP) – located in the submucosal layer, beneath the mucosal layer.

The enteric neurons are characterised by high morphological, functional and neurochemical diversity [11]. Till now, several dozens of substances acting as neurotransmitters, neuromodulators, enzymes or transporters have been described in the enteric neurons [12–14]. One of these substances is cocaine- and amphetamine-regulated transcript (CART) peptide [11].

CART peptide occurs in both the central and peripheral nervous system of various mammal species, including humans, and it is considered to be one of the most important factors involved in the regulation of food intake and anorexigenic actions [14–16]. Moreover, CART plays a part in the development of the nervous system and has neuroprotective properties [17,18]. CART has also been identified in the enteric nervous system in all the enteric plexuses in various segments of the gastrointestinal tract of a diversity of mammal species [14]. CART in the intestine is probably involved in the regulation of peristalsis and secretory activity, but not through a direct effect on the intestinal wall but through the CNS [19]. Locally, the CART-positive enteric neurons are also involved in the plastic modulations of the enteric neurons under the impact of physiological and pathological stimuli [20,21]. However, the exact roles of CART in the intestine still remain unexplained.

The enteric neurons can respond to a wide range of physiological and pathological factors [22–24]. These changes concern the structure and functions of neurons but primarily manifest themselves in a change in their neurochemical characteristics and fluctuation in intraneuronal synthesis, transport and secretion of active factors [24–26]. The majority of studies focus on the changes in the ENS under pathological stimuli, including gastrointestinal and systemic diseases, inflammatory processes and toxic substances in the food [21,22,27–30]. The ENS can also change under physiological factors, including age, gender or diet, but the knowledge about these issues is much more limited [31–35].

Previous results have described changes in the number of enteric neurons containing CART exposed to diverse pathological factors, which strongly suggests that CART–positive neurons in the gastrointestinal tract take part in adaptive and/or protective processes [21,36–38]. On the other hand, information about the participation of CART–positive enteric neurons in adaptive processes during aging is extremely scanty and is limited to only one study, which describes age-dependent changes in the degree of co-localisation of calcitonin gene-related peptide and CART in the same enteric neurons [39]. However, to date, the influence of age and/or gender on the percentage of enteric neurons containing CART has not been studied at all.

It should be pointed out that the choice of the animal species and segment of the digestive tract was not random. It is relatively well known that the ENS in the domestic pig shows great similarities to human ENS in terms of organisation, biochemistry and neurochemical characterisation of neurons [40,41]. Therefore, physiological and pathological processes and neuroplasticity reactions in the enteric neurons are similar in both these mammal species, and the domestic pig is considered to be an optimal animal model for studying the human ENS [42,43]. In turn, the descending colon is a segment of the digestive tract where many diseases can develop. In many pathological processes occurring in the descending colon, e.g., Crohn’s disease and cancer, changes in the ENS have been noted [44–46], and the risk of these processes often depends both on the age and gender of individuals [29].

Given the above, the aim of the present study was to investigate for the first time the correlation between age and gender and the number of neurons containing CART in the ENS localised in the wall of the descending colon of the domestic pig. The results obtained in the present work may contribute to a better understanding of the roles of CART in neuroplasticity processes occurring during the development and aging of the ENS.

## Materials and methods

The investigation was performed on the tissues collected from domestic pigs slaughtered at commercial slaughterhouses using a typical method of slaughter (carbon dioxide). All experimental protocols were approved by the Local Ethical Committee for Animal Experiments in Olsztyn working at the University of Warmia and Mazury in Olsztyn, according to Act for the Protection of Animals for Scientific or Educational Purposes of 15 January 2015 (Official Gazette 2015, No. 266), applicable in the Republic of Poland (based on the consent No 28/2013 of 22 May 2013 and 65/2013/DLZ of 27 Nov 2013). During this experiment, all methods were carried out in accordance with relevant guidelines and European and Polish regulations. Moreover, the study was carried out in compliance with the ARRIVE guidelines.

The fragments of the descending colon with a length of about 2 cm and located about 30 cm before the anus were collected from 20 animals divided into four groups (n = 5 in each group): 1) YF group – young female pigs before puberty (about 10 weeks old); 2) AF group – adult female pigs after puberty (7–8 months old); 3) YM group – young male pigs before puberty (about 10 weeks old) and 4) AM – adult male pigs after puberty (7–8 months old). Male pigs were castrated at the age of about one week.

The intestinal fragments were collected immediately after the death of the animals during bowel removal in post-slaughter processing. Just after sampling, tissues were put into a solution of 4% buffered paraformaldehyde (pH 7.4) for 1 h to perform immersive fixation. The fragments of the intestine were then rinsed in a phosphate buffer (0.1 M, pH 7.4) for three days at 5 °C with a daily exchange of the buffer. After this period, tissues were put in an 18% phosphate-buffered sucrose solution and stored at 5 °C for at least three weeks until further study. After this period, the fragments of the colon were frozen at − 22 °C, cut perpendicular to the intestinal lumen into 14-µm-thick sections with a microtome (Microm, HM 525, Walldorf, Germany) and mounted on the microscopic slides.

Intestinal sections were subjected to the typical double immunofluorescence method described previously by Makowska and Gonkowski [39]. In brief, the method consisted of the following stages: 1) drying the sections at room temperature for 45 min; 2) incubation with “blocking” solution (10% goat serum, 0.1% bovine serum albumin (BSA), 0.01% NaN_3_, Triton X-100, and thimerosal in PBS) for 1 h to prevent non-specific staining; 3) overnight incubation with the mixture of two antibodies: mouse antibody directed against protein gene product 9.5 (PGP 9.5, used as a pan-neuronal marker obtained from Biogenesis Ltd., Poole, UK, (working dilution 1:1000) and rabbit antibody against CART peptide purchased from Phoenix Pharmaceuticals, Inc., Burlingame, CA, USA (working dilution 1:16000); 4) one-hour incubation with a mixture of species-specific secondary antisera: donkey anti-mouse IgG conjugated with Alexa fluor 488 and donkey anti-rabbit IgG conjugated with Alexa fluor 546 (both from Invitrogen, Carlsbad, CA, USA, both in a working dilution of 1:1000); 5) covering the sections with buffered glycerol and coverslips. The information about antibodies used I the present study is summarized in Table 1.

**Table 1:** The list of primary and secondary antibodies used in this study.

Primary Antibodies					
**Antigen**	**Code**	**Species**	**Working Dilution**		**Supplier**
PGP 9.5	7863-2004	Mouse	1:1000		Biogenesis Ltd., Poole, UK
CART	H-003–61	Rabbit	1:16000		Phoenix Pharmaceuticals, Inc., Burlingame, CA, USA
**Secondary Antibodies**					
**Reagents**			**Code**	**Working** **Dilution**	**Supplier**
Alexa fluor 488 donkey anti-mouse IgG			R37114	1:1000	Invitrogen, Carlsbad, CA, USA
Alexa fluor 546 donkey anti-rabbit IgG			A10040	1:1000	Invitrogen,

All of the above-mentioned stages were performed at room temperature. Stages no. 2,3, and 4 were performed in a humid chamber. Between each stage of labelling, colon sections were rinsed in PBS for 30 min with a PBS change every 10 min. Moreover 4′,6-diamidino-2-phenylindole (DAPI) obtained from Sigma obtained from Sigma-Aldirch (St Louis, MO, USA) in working dilution 1 µ /ml was used to visualise cell nuclei.

Checking the specificity of the staining performed consisted of typical testing, including 1) pre-absorption of antibodies with appropriate antigens: PGP 9.5 (AbD Serotec, Kidlington, UK) or CART (Phoenix Pharmaceuticals, Burlingame, CA, USA) at a concentration of 20 μg/mL, overnight at room temperature; 2) omission test and 3) replacement of primary antibodies by non–immune sera. Negative staining was obtained during the pre-absorption of antibodies after applying the above tests.

Intestinal fragments were analysed using an immunofluorescence microscope BX51 (Olympus, Tokyo, Japan) equipped with appropriate filters. To establish the percentage of enteric neurons containing CART, at least 500 cells immunoreactive to PGP 9.5 located in each type of enteric ganglia were evaluated for the presence of CART in each animal included in the study. The results were presented in percentages, and the number of analysed PGP 9.5-positive cells was considered 100%. To prevent double counting the same neurons, sections of the descending colon included in the analysis were located at least 200 μm from each other. The obtained results were presented as mean values ±  SEM. A statistical analysis was performed with a T-Student test using GraphPad Prism version 9.2.0 (GraphPad Software, San Diego, California, USA). Differences were considered statistically significant at P < 0.05.

## Results

Neurons containing CART were found in all types of colonic enteric plexuses in all groups of animals included in the study.

**Fig 1:**
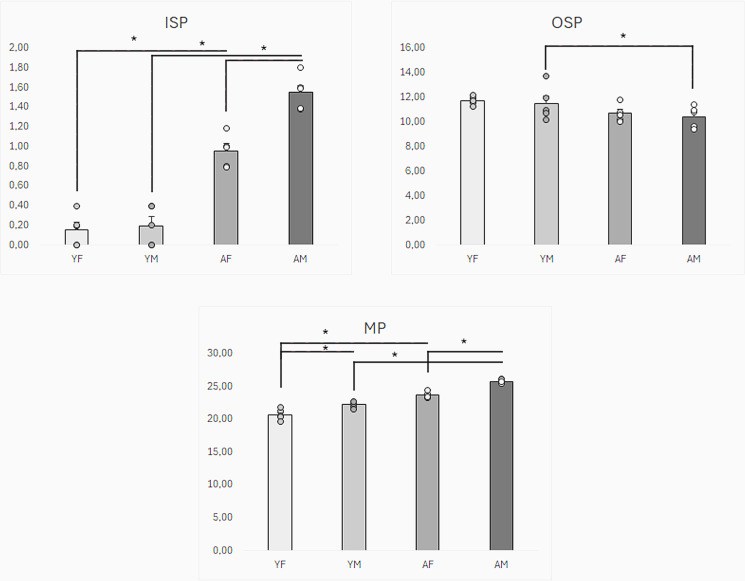
Graphical comparison of the number of nerve cells immunoreactive to protein gene product (PGP 9.5) – used here as a pan-neuronal marker and cocaine- and amphetamine-regulated peptide (CART) in inner submucous (ISP), outer submucous (OSP) and myenteric plexus (MP) of porcine colon between young females (YF), young males (YM), adult females (AF) and adult males (AM). Statistically significant differences between groups are marked with * .

**Fig 2:**
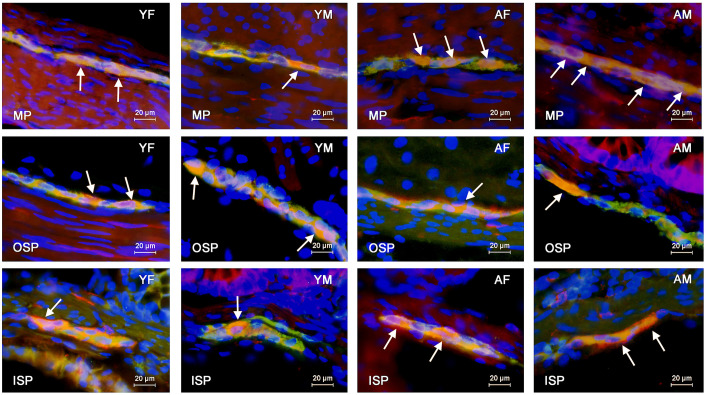
Distribution pattern of nerve cells immunoreactive to protein gene-product 9.5 (PGP 9.5) – used as a pan-neuronal marker – and cocaine- and amphetamine-regulated peptide (CART) in the myenteric (MP), outer submucous (OSP) and inner submucous (ISP) plexuses of porcine descending colon in young females (YF), young males (YM), adult females (AF) and adult males (AM). The images are the result of the overlap of both stainings. The arrows indicate neurons immunoreactive to both – PGP 9.5 and the other neuronal active substance studied.

The largest percentage of CART-positive neurons was observed in the MP. In young female pigs, the percentage of such neurons amounted to 22.3 ±  0.2% of all cells immunoreactive to PGP 9.5 and was statistically significantly higher than the value noted in young male pigs, where the percentage of CART-positive cells achieved the level of 20.7 ±  0.4%. Moreover, the number of CART-like immunoreactive neurons in the MP increased with the growth and maturation of the organism, and this increase was more visible in male pigs. In adult pigs, the percentage of neurons containing CART amounted to 23.7 ±  0.2% and 25.8 ±  0.1% in females and males, respectively. These values were statistically significantly higher than these values in young animals of the appropriate gender. Moreover, contrary to young animals, the percentage of CART-positive cells noted in adult males was statistically significantly higher than that noted in females (Fig 1, Fig 2 Table 2).

**Table 2:** The percentage (mean ±  SEM) of enteric neurons containing CART in the porcine descending colon under physiological conditions in young (10-week-old) females and males and adult (7–8-month-old) females and males.

	YF	YM	AF	AM
**MP**	22.3 ± 0.22^ad^	20.7 ± 0.4^ac^	23.7 ± 0.2^bd^	25.8 ± 0.1^bc^
**OSP**	11.5 ± 0.6	11.7 ± 0.2^c^	10.7 ± 0.3	10.4 ± 0.4^c^
**ISP**	0.2 ± 0.1^d^	0.2 ± 0.1^c^	1.0 ± 0.1^bd^	1.6 ± 0.1^bc^

MP: myenteric plexus; OSP: outer submucous plexus; ISP: inner submucous plexus; YF – young female, YM – young male; AF – adult female, AM – adult male. Statistically significant (*P* ≤ 0.05) differences are marked as follows: between young females and males with ^a^, between adult females and males with ^b^, between young and adult males with ^c^, between young and adult females with ^d^.

A much smaller percentage of CART-positive neurons was observed in the OSP. In the young animals, these values amounted to 11.5 ±  0.6% and 11.7 ±  0.2% of all PGP 9.5-positive cells in young females and males, respectively. There were no statistically significant differences between genders in young animals. In adult females, the percentage of CART-positive cells amounted to 10.7 ±  0.3% and did not differ statistically significantly from that observed in young pigs of this gender. In turn, in adult males, the percentage of neurons immunoreactive to CART achieved the level of 10.4 ±  0.4% and was statistically significantly lower than that found in young males. There were no statistically significant differences between genders in adult animals (Fig 1).

In the ISP, CART-positive neurons were the least numerous. In young animals, the percentage of such neurons achieved the level of 0.2 ±  0.1% both in females and males. These values increased slightly with increasing age but still oscillated only around 1% of all PGP 9.5 – positive cells. In adult animals, the percentage of neurons containing CART amounted to 1.0 ±  0.1% and 1.6 ±  0.1%. Both in adult females and males, the values were statistically significantly higher than those noted in young animals of the same gender. Moreover, the difference in the percentage of CART-positive neurons between adult females and males was statistically significant (Fig 1). The results obtained in the present study are summarised in Table 2.

## Discussion

During the present study, neurons containing CART have been noted in all types of enteric plexuses localised in the descending colon of both young and adult animals, which may confirm the multidirectional roles of this peptide in the digestive tract suggested by previous studies [17,28,47]. Both the present observation and previous investigations have shown the largest population of CART-positive neurons in the MP, which confirms that CART is a factor that, first of all, regulates intestinal motility [17,28,48,49]. In turn, the small number of CART-containing cells in the ISP suggests that this peptide plays a relatively minor role in intestinal secretion, although some previous studies have described the possibility of CART participation in the regulation of the gastrointestinal glands’ activity [50,51]. Moreover, the present study confirms intra-species differences in the distribution of CART in the enteric neurons [17,20,21,48]. However, it should be pointed out, that there are also clear differences in the percentage of CART-positive neurons between the present study and previous investigations performed on juvenile domestic pigs [14,48]. This fact strongly suggests that the synthesis and distribution of this peptide in the enteric neurons may depend on the race of animals, food and/or other not quite clear environmental factors.

It is commonly known that the ENS is a complex structure that may change under the influence of many pathological and physiological factors [22,24]. These changes include neuronal morphology, functions and neurochemical characterisation and reflect adaptive processes in response to acting stimuli [43,52,53]. Contrary to the influence of pathological and toxic factors on the ENS, the knowledge about changes in the enteric neurons caused by physiological factors (especially by maturation and aging) is rather scanty, but it is known that these changes may manifest themselves by modification of the number and shape of neurons and their ability to synthesise active substances [35,54,55].

The results obtained in the present study confirmed age-dependent changes in the population of the enteric neurons containing CART in the porcine colon. It should be underlined that maturation-induced changes in the intestine are multidirectional and concern the muscular layer, intramural blood vessels and intestinal secretory activity [56,57]. Moreover, maturation and aging directly affect the ENS, changing the length, number and appearance of axons and dendrites of the enteric neurons, as well as nerve electrophysiological properties and organisation of synapses [25,39].

The present results strongly suggest the participation of CART in adaptive processes connected with the development, maturation and aging of the large intestine. It is all the more likely that CART has been described as a factor participating in the development and differentiation of neuronal cells and synaptic connections within the central nervous system [58,59]. In light of this research, it is highly probable that CART may perform similar functions within the ENS. Age-dependent changes in the number of CART-positive neurons may also result from interactions between intestinal bacterial flora and the ENS. This thesis is supported by the understanding that the maturation process significantly affects the composition of the intestinal flora [60]. Additionally, research has shown that the microbiota can modulate the enteric neurons that contain CART [61].

The next reason for the increase in the percentage of enteric CART-positive neurons in older animals may be connected with the neurotrophic and protective functions of this peptide, as suggested by previous studies [62,63]. It is commonly known that throughout life, the intestinal wall is affected by many mechanical, chemical and stress stimuli [56,57,64]. Such a situation can lead to the development of inflammatory processes and various intestinal diseases in later life [65,66]. On the other hand, it is known that the ENS shows protective and regenerative abilities and protects the gastrointestinal tract in pathological states [4,10,43,53]. Therefore, the increase in the percentage of neurons containing CART in the MP and ISP noted in the present study may be connected with these processes because CART in some previous studies has been described as a protective factor [19,62,63,67]. However, in light of the facts mentioned above and noted in the present observation, the slight decrease in the number of CART–positive cells in the OSP of adult males in comparison to young males is not clear.

During the present study, gender-dependent differences in the percentage of CART-positive neurons in the colonic ENS were also noted, and these differences were more visible in adult animals. This fact confirms that the neurochemical characterisation of the enteric neurons depends on gender. Previous knowledge about the influence of gender on the organisation of the ENS is rather limited [39,68,69]. It is known that receptors for sex hormones are widely distributed in the gastrointestinal tract, including the enteric neurons and glial cells [70]. Previous studies have also reported that sex hormones affect the ENS. It was found that stimulation of oestrogen receptor β leads to neuroprotective reactions involving enteric glial cell proliferation and neuronal differentiation [71].

Nevertheless, previous reports concerning gender-dependent differences within the ENS are inconclusive. Some of them have described such differences in the reaction of the ENS to some pathological and stress stimuli [72], as well as in the neurochemical characterisation of the enteric neurons [39]. In turn, other studies have not reported diversities in the organisation of the ENS between males and females [73]. The results obtained in the present study have confirmed gender-dependent differences in CART distribution in the enteric neurons. This is in agreement with previous reports on the central nervous system, where the knowledge about CART is better developed and gender-dependent diversity in the distribution and functions of structures containing CART have been reported [74].

## Conclusions

To sum up, the results obtained in the present study have shown that the percentage of the neurons containing CART in the colonic ENS of the domestic pig depends not only on the type of the enteric plexus but also on the age of the animals. The presence of CART-positive neurons in all types of enteric plexuses confirms the multidirectional roles of CART in the regulation of large intestine functions. In turn, age-dependent changes in the number of CART-positive enteric neurons strongly suggest that this peptide plays a part in the processes connected with the development, maturation, and/or aging of the ENS and gastrointestinal tract. The results have also shown gender-dependent diversities in the distribution of CART in the enteric neurons. This fact shows that CART-positive neurons are influenced by sex hormones and suggests various exact functions of this peptide in the male and female gastrointestinal tract. However, it should be pointed out that clarification of all issues related to the exact functions of CART in the regulation of the functioning of the ENS in the large intestine requires further comprehensive research.

## References

[1] Gabella G. Innervation of the gastrointestinal tract. Int Rev Cytol. 1979;59:129–93. doi: 10.1016/s0074-7696(08)61662-9 226496

[2] Rao M, Gershon MD. Enteric nervous system development: what could possibly go wrong?. Nat Rev Neurosci. 2018;19(9):552–65. doi: 10.1038/s41583-018-0041-0 30046054 PMC6261281

[3] Furness JB, Callaghan BP, Rivera LR, Cho H-J. The enteric nervous system and gastrointestinal innervation: integrated local and central control. Adv Exp Med Biol. 2014;817:39–71. doi: 10.1007/978-1-4939-0897-4_3 24997029

[4] Makowska K. Chemically induced inflammation and nerve damage affect the distribution of vasoactive intestinal polypeptide-like immunoreactive (VIP-LI) nervous structures in the descending colon of the domestic pig. Neurogastroenterol Motil. 2018;30(11):e13439. doi: 10.1111/nmo.13439 30109906

[5] Schneider S, Wright CM, Heuckeroth RO. Unexpected Roles for the Second Brain: Enteric Nervous System as Master Regulator of Bowel Function. Annu Rev Physiol. 2019;81:235–59. doi: 10.1146/annurev-physiol-021317-121515 30379617

[6] Morikawa S, Komuro T. Distribution of myenteric NO neurons along the guinea-pig esophagus. J Auton Nerv Syst. 1998;74(2–3):91–9. doi: 10.1016/s0165-1838(98)00131-3 9915623

[7] Reiche D, Michel K, Pfannkuche H, Schemann M. Projections and neurochemistry of interneurones in the myenteric plexus of the guinea-pig gastric corpus. Neurosci Lett. 2000;295(3):109–12. doi: 10.1016/s0304-3940(00)01617-7 11090986

[8] Zhang G-Q, Yang S, Li X-S, Zhou D-S. Expression and possible role of IGF-IR in the mouse gastric myenteric plexus and smooth muscles. Acta Histochem. 2014;116(5):788–94. doi: 10.1016/j.acthis.2014.01.011 24630395

[9] Zimmermann J, Neuhuber WL, Raab M. Homer1 (VesL-1) in the rat esophagus: focus on myenteric plexus and neuromuscular junction. Histochem Cell Biol. 2017;148(2):189–206. doi: 10.1007/s00418-017-1555-7 28337539

[10] Gonkowski S, Rowniak M, Wojtkiewicz J. Zinc Transporter 3 (ZnT3) in the Enteric Nervous System of the Porcine Ileum in Physiological Conditions and during Experimental Inflammation. Int J Mol Sci. 2017;18(2):338. doi: 10.3390/ijms18020338 28178198 PMC5343873

[11] Brehmer A. Classification of human enteric neurons. Histochem Cell Biol. 2021;156(2):95–108. doi: 10.1007/s00418-021-02002-y 34170401 PMC8397665

[12] Timmermans JP, Scheuermann DW, Stach W, Adriaensen D, De Groodt-Lasseel MH. Functional morphology of the enteric nervous system with special reference to large mammals. Eur J Morphol. 1992;30(2):113–22. 1457247

[13] Furness JB. Types of neurons in the enteric nervous system. J Auton Nerv Syst. 2000;81(1–3):87–96. doi: 10.1016/s0165-1838(00)00127-2 10869706

[14] Makowska K, Gonkowski S. Cocaine- and Amphetamine-Regulated Transcript (CART ) Peptide in Mammals Gastrointestinal System – A Review. Annals of Animal Science. 2017;17(1):3–21. doi: 10.1515/aoas-2016-0014

[15] Thim L, Nielsen PF, Judge ME, Andersen AS, Diers I, Egel-Mitani M, et al. Purification and characterisation of a new hypothalamic satiety peptide, cocaine and amphetamine regulated transcript (CART), produced in yeast. FEBS Lett. 1998;428(3):263–8. doi: 10.1016/s0014-5793(98)00543-2 9654146

[16] Jensen PB, Kristensen P, Clausen JT, Judge ME, Hastrup S, Thim L, et al. The hypothalamic satiety peptide CART is expressed in anorectic and non-anorectic pancreatic islet tumors and in the normal islet of Langerhans. FEBS Lett. 1999;447(2–3):139–43. doi: 10.1016/s0014-5793(99)00291-4 10214934

[17] Ekblad E. CART in the enteric nervous system. Peptides. 2006;27(8):2024–30. doi: 10.1016/j.peptides.2005.12.015 16759747

[18] Mao P, Ardeshiri A, Jacks R, Yang S, Hurn PD, Alkayed NJ. Mitochondrial mechanism of neuroprotection by CART. Eur J Neurosci. 2007;26(3):624–32. doi: 10.1111/j.1460-9568.2007.05691.x 17634068 PMC2582219

[19] Ekblad E, Kuhar M, Wierup N, Sundler F. Cocaine- and amphetamine-regulated transcript: distribution and function in rat gastrointestinal tract. Neurogastroenterol Motil. 2003;15(5):545–57. doi: 10.1046/j.1365-2982.2003.00437.x 14507354

[20] Oponowicz A, Kozłowska A, Gonkowski S, Godlewski J, Majewski M. Changes in the Distribution of Cocaine- and Amphetamine-Regulated Transcript-Containing Neural Structures in the Human Colon Affected by the Neoplastic Process. Int J Mol Sci. 2018;19(2):414. doi: 10.3390/ijms19020414 29385033 PMC5855636

[21] Makowska K, Fagundes KRC, Gonkowski S. Influence of bisphenol A and its analog bisphenol S on cocaine- and amphetamine-regulated transcript peptide-positive enteric neurons in the mouse gastrointestinal tract. Front Mol Neurosci. 2023;16:1234841. doi: 10.3389/fnmol.2023.1234841 37675141 PMC10477371

[22] Lomax AE, Fernández E, Sharkey KA. Plasticity of the enteric nervous system during intestinal inflammation. Neurogastroenterol Motil. 2005;17(1):4–15. doi: 10.1111/j.1365-2982.2004.00607.x 15670258

[23] Sharkey KA, Mawe GM. The enteric nervous system. Physiol Rev. 2023;103(2):1487–564. doi: 10.1152/physrev.00018.2022 36521049 PMC9970663

[24] Furness JB. The enteric nervous system: normal functions and enteric neuropathies. Neurogastroenterol Motil. 2008;20 Suppl 1:32–8. doi: 10.1111/j.1365-2982.2008.01094.x 18402640

[25] Saffrey MJ. Cellular changes in the enteric nervous system during ageing. Dev Biol. 2013;382(1):344–55. doi: 10.1016/j.ydbio.2013.03.015 23537898

[26] Rytel L, Wojtkiewicz J, Snarska A, Mikołajczyk A. Changes in the Neurochemical Characterization of Enteric Neurons in the Porcine Duodenum After Administration of Low-Dose Salmonella Enteritidis Lipopolysaccharides. J Mol Neurosci. 2021;71(8):1556–66. doi: 10.1007/s12031-019-01473-y 31939106

[27] Margolis KG, Gershon MD. Enteric Neuronal Regulation of Intestinal Inflammation. Trends Neurosci. 2016;39(9):614–24. doi: 10.1016/j.tins.2016.06.007 27450201 PMC5002370

[28] Gonkowski S, Gajęcka M, Makowska K. Mycotoxins and the Enteric Nervous System. Toxins (Basel). 2020;12(7):461. doi: 10.3390/toxins12070461 32707706 PMC7404981

[29] Niesler B, Kuerten S, Demir IE, Schäfer K-H. Disorders of the enteric nervous system - a holistic view. Nat Rev Gastroenterol Hepatol. 2021;18(6):393–410. doi: 10.1038/s41575-020-00385-2 33514916

[30] Marinsek GP, Choueri PKG, Choueri RB, de Souza Abessa DM, Gonçalves ARN, Bortolotto LB, et al. Integrated analysis of fish intestine biomarkers: Complementary tools for pollution assessment. Mar Pollut Bull. 2022;178:113590. doi: 10.1016/j.marpolbul.2022.113590 35367694

[31] De Britto Mari R, Clebis NK, Gagliardo KM, Guimarães JP, Stabille SR, De Mello Germano R, et al. Effects of exercise on the morphology of the myenteric neurons of the duodenum of Wistar rats during the ageing process. Anat Histol Embryol. 2008;37(4):289–95. doi: 10.1111/j.1439-0264.2008.00843.x 18384460

[32] Mari R de B, Stabille SR, de Faria HG, Pereira JNB, Guimarães JP, Marinsek GP, et al. Balanced Caloric Restriction Minimizes Changes Caused by Aging on the Colonic Myenteric Plexus. J Diet Suppl. 2018;15(3):285–99. doi: 10.1080/19390211.2017.1341446 28759281

[33] Pereira JNB, Mari RB, Stabille SR, de Faria HG, Mota TFM, Ferreira WM. Benefits of caloric restriction in the myenteric neuronal plasticity in aging rats. An Acad Bras Cienc. 2014;86(3):1471–81. doi: 10.1590/0001-3765201420130052 25211115

[34] Bernard CE, Gibbons SJ, Gomez-Pinilla PJ, Lurken MS, Schmalz PF, Roeder JL, et al. Effect of age on the enteric nervous system of the human colon. Neurogastroenterol Motil. 2009;21(7):746-e46. doi: 10.1111/j.1365-2982.2008.01245.x 19220755 PMC2776702

[35] Nguyen TT, Baumann P, Tüscher O, Schick S, Endres K. The Aging Enteric Nervous System. Int J Mol Sci. 2023;24(11):9471. doi: 10.3390/ijms24119471 37298421 PMC10253713

[36] Gonkowski S, Burliński P, Szwajca P, Całka J. Changes in Cocaine- and Amphetamine-Regulated Transcript-Like Immunoreactive (CART-LI) Nerve Structures of the Porcine Descending Colon During Proliferative Enteropathy. Bulletin of the Veterinary Institute in Pulawy. 2012;56(2):199–203. doi: 10.2478/v10213-012-0036-y

[37] Bulc M, Całka J, Palus K. Effect of Streptozotocin-Inducted Diabetes on the Pathophysiology of Enteric Neurons in the Small Intestine Based on the Porcine Diabetes Model. Int J Mol Sci. 2020;21(6):2047. doi: 10.3390/ijms21062047 32192078 PMC7139978

[38] Makowska K, Gonkowski S, Zielonka L, Dabrowski M, Calka J. T2 Toxin-Induced Changes in Cocaine- and Amphetamine-Regulated Transcript (CART)-Like Immunoreactivity in the Enteric Nervous System Within Selected Fragments of the Porcine Digestive Tract. Neurotox Res. 2017;31(1):136–47. doi: 10.1007/s12640-016-9675-8 27738989 PMC5209419

[39] Makowska K, Gonkowski S. Age and Sex-Dependent Differences in the Neurochemical Characterization of Calcitonin Gene-Related Peptide-Like Immunoreactive (CGRP-LI) Nervous Structures in the Porcine Descending Colon. Int J Mol Sci. 2019;20(5):1024. doi: 10.3390/ijms20051024 30818742 PMC6429317

[40] Douglas WR. Of pigs and men and research: a review of applications and analogies of the pig, sus scrofa, in human medical research. Space Life Sci. 1972;3(3):226–34. doi: 10.1007/BF00928167 4556756

[41] Patterson JK, Lei XG, Miller DD. The pig as an experimental model for elucidating the mechanisms governing dietary influence on mineral absorption. Exp Biol Med (Maywood). 2008;233(6):651–64. doi: 10.3181/0709-MR-262 18408137

[42] Verma N, Rettenmeier AW, Schmitz-Spanke S. Recent advances in the use of Sus scrofa (pig) as a model system for proteomic studies. Proteomics. 2011;11(4):776–93. doi: 10.1002/pmic.201000320 21229584

[43] Makowska K, Gonkowski S. Changes in the Enteric Neurons Containing Selected Active Substances in the Porcine Descending Colon after the Administration of Bisphenol A (BPA). Int J Environ Res Public Health. 2022;19(23):16187. doi: 10.3390/ijerph192316187 36498260 PMC9739061

[44] Belai A, Boulos PB, Robson T, Burnstock G. Neurochemical coding in the small intestine of patients with Crohn’s disease. Gut. 1997;40(6):767–74. doi: 10.1136/gut.40.6.767 9245931 PMC1027202

[45] Godlewski J, Kmiec Z. Colorectal Cancer Invasion and Atrophy of the Enteric Nervous System: Potential Feedback and Impact on Cancer Progression. Int J Mol Sci. 2020;21(9):3391. doi: 10.3390/ijms21093391 32403316 PMC7247003

[46] Magalhães HIR, Castelucci P. Enteric nervous system and inflammatory bowel diseases: Correlated impacts and therapeutic approaches through the P2X7 receptor. World J Gastroenterol. 2021;27(46):7909–24. doi: 10.3748/wjg.v27.i46.7909 35046620 PMC8678817

[47] Wierup N, Gunnarsdóttir A, Ekblad E, Sundler F. Characterisation of CART-containing neurons and cells in the porcine pancreas, gastro-intestinal tract, adrenal and thyroid glands. BMC Neurosci. 2007;8:51. doi: 10.1186/1471-2202-8-51 17625001 PMC1934373

[48] Gonkowski S, Burliński P, Skobowiat C, Majewski M, Arciszewski MB, Radziszewski P, et al. Distribution of cocaine- and amphetamine-regulated transcript-like immunoreactive (CART-LI) nerve structures in the porcine large intestine. Acta Vet Hung. 2009;57(4):509–20. doi: 10.1556/AVet.57.2009.4.5 19897455

[49] Tebbe JJ, Ortmann E, Schumacher K, Mönnikes H, Kobelt P, Arnold R, et al. Cocaine- and amphetamine-regulated transcript stimulates colonic motility via central CRF receptor activation and peripheral cholinergic pathways in fed, conscious rats. Neurogastroenterol Motil. 2004;16(4):489–96. doi: 10.1111/j.1365-2982.2004.00561.x 15306004

[50] Okumura T, Yamada H, Motomura W, Kohgo Y. Cocaine-amphetamine-regulated transcript (CART) acts in the central nervous system to inhibit gastric acid secretion via brain corticotropin-releasing factor system. Endocrinology. 2000;141(8):2854–60. doi: 10.1210/endo.141.8.7588 10919272

[51] Shcherbina L, Lindqvist A, Thorén Fischer A-H, Ahlqvist E, Zhang E, Falkmer SE, et al. Intestinal CART is a regulator of GIP and GLP-1 secretion and expression. Mol Cell Endocrinol. 2018;476:8–16. doi: 10.1016/j.mce.2018.04.002 29627317

[52] Werner CM, Willing LB, Goudsward HJ, McBride AR, Stella SL Jr, Holmes GM. Plasticity of colonic enteric nervous system following spinal cord injury in male and female rats. Neurogastroenterol Motil. 2023;35(11):e14646. doi: 10.1111/nmo.14646 37480186 PMC11298951

[53] Vasina V, Barbara G, Talamonti L, Stanghellini V, Corinaldesi R, Tonini M, et al. Enteric neuroplasticity evoked by inflammation. Autonomic Neuroscience. 2006;126–127:264–72. doi: 10.1016/j.autneu.2006.02.025 16624634

[54] Foong JPP. Postnatal Development of the Mouse Enteric Nervous System. Adv Exp Med Biol. 2016;891:135–43. doi: 10.1007/978-3-319-27592-5_13 27379641

[55] van Haver ER, de Vooght L, Oste M, Sangild PT, Thymann T, Weyns ALM, et al. Postnatal and diet-dependent increases in enteric glial cells and VIP-containing neurones in preterm pigs. Neurogastroenterol Motil. 2008;20(9):1070–9. doi: 10.1111/j.1365-2982.2008.01160.x 18643892

[56] Chin AM, Hill DR, Aurora M, Spence JR. Morphogenesis and maturation of the embryonic and postnatal intestine. Semin Cell Dev Biol. 2017;66:81–93. doi: 10.1016/j.semcdb.2017.01.011 28161556 PMC5487846

[57] Neu J. Gastrointestinal maturation and feeding. Semin Perinatol. 2006;30(2):77–80. doi: 10.1053/j.semperi.2006.02.007 16731281

[58] Subhedar NK, Nakhate KT, Upadhya MA, Kokare DM. CART in the brain of vertebrates: circuits, functions and evolution. Peptides. 2014;54:108–30. doi: 10.1016/j.peptides.2014.01.004 24468550

[59] Abrahám H, Orsi G, Seress L. Ontogeny of cocaine- and amphetamine-regulated transcript (CART) peptide and calbindin immunoreactivity in granule cells of the dentate gyrus in the rat. Int J Dev Neurosci. 2007;25(5):265–74. doi: 10.1016/j.ijdevneu.2007.05.008 17616293

[60] Enck P, Zimmermann K, Rusch K, Schwiertz A, Klosterhalfen S, Frick JS. The effects of maturation on the colonic microflora in infancy and childhood. Gastroenterol Res Pract. 2009;2009:752401. doi: 10.1155/2009/752401 19763278 PMC2744901

[61] Muller PA, Matheis F, Schneeberger M, Kerner Z, Jové V, Mucida D. Microbiota-modulated CART+ enteric neurons autonomously regulate blood glucose. Science. 2020;370(6514):314–21. doi: 10.1126/science.abd6176 32855216 PMC7886298

[62] Risold PY, Bernard-Franchi G, Collard C, Jacquemard C, La Roche A, Griffond B. Ontogenetic expression of CART-peptides in the central nervous system and the periphery: a possible neurotrophic role?. Peptides. 2006;27(8):1938–41. doi: 10.1016/j.peptides.2005.10.026 16725226

[63] Zhang M, Han L, Xu Y. Roles of cocaine- and amphetamine-regulated transcript in the central nervous system. Clin Exp Pharmacol Physiol. 2012;39(6):586–92. doi: 10.1111/j.1440-1681.2011.05642.x 22077697

[64] Kulkarni S, Saha M, Slosberg J, Singh A, Nagaraj S, Becker L, et al. Age-associated changes in lineage composition of the enteric nervous system regulate gut health and disease. Elife. 2023;12:RP88051. doi: 10.7554/eLife.88051 38108810 PMC10727506

[65] Thevaranjan N, Puchta A, Schulz C, Naidoo A, Szamosi JC, Verschoor CP, et al. Age-Associated Microbial Dysbiosis Promotes Intestinal Permeability, Systemic Inflammation, and Macrophage Dysfunction. Cell Host Microbe. 2017;21(4):455-466.e4. doi: 10.1016/j.chom.2017.03.002 28407483 PMC5392495

[66] Amersi F, Agustin M, Ko CY. Colorectal cancer: epidemiology, risk factors, and health services. Clin Colon Rectal Surg. 2005;18(3):133–40. doi: 10.1055/s-2005-916274 20011296 PMC2780097

[67] Jia J, Chen X, Zhu W, Luo Y, Hua Z, Xu Y. CART protects brain from damage through ERK activation in ischemic stroke. Neuropeptides. 2008;42(5–6):653–61. doi: 10.1016/j.npep.2008.05.006 18644622

[68] Ameku T, Beckwith H, Blackie L, Miguel-Aliaga I. Food, microbes, sex and old age: on the plasticity of gastrointestinal innervation. Curr Opin Neurobiol. 2020;62:83–91. doi: 10.1016/j.conb.2019.12.004 32028080 PMC7294223

[69] Million M, Larauche M. Stress, sex, and the enteric nervous system. Neurogastroenterol Motil. 2016;28(9):1283–9. doi: 10.1111/nmo.12937 27561694 PMC5003424

[70] Liu JYH, Lin G, Fang M, Rudd JA. Localization of estrogen receptor ERα, ERβ and GPR30 on myenteric neurons of the gastrointestinal tract and their role in motility. Gen Comp Endocrinol. 2019;272:63–75. doi: 10.1016/j.ygcen.2018.11.016 30502347

[71] Nie X, Xie R, Tuo B. Effects of Estrogen on the Gastrointestinal Tract. Dig Dis Sci. 2018;63(3):583–96. doi: 10.1007/s10620-018-4939-1 29387989

[72] Medland JE, Pohl CS, Edwards LL, Frandsen S, Bagley K, Li Y, et al. Early life adversity in piglets induces long-term upregulation of the enteric cholinergic nervous system and heightened, sex-specific secretomotor neuron responses. Neurogastroenterol Motil. 2016;28(9):1317–29. doi: 10.1111/nmo.12828 27134125 PMC5002263

[73] Korolkiewicz R, Sliwiński W, Rekowski P, Halama A, Mucha P, Szczurowicz A, et al. Contractile action of galanin analogues on rat isolated gastric fundus strips is modified by tachyphylaxis to substance P. Pharmacol Res. 1996;33(6):361–5. doi: 10.1006/phrs.1996.0050 8971959

[74] Fagergren P, Hurd YL. Mesolimbic gender differences in peptide CART mRNA expression: effects of cocaine. Neuroreport. 1999;10(16):3449–52. doi: 10.1097/00001756-199911080-00034 10599860

